# Supplementary material for: The mediating effects of perceived social support and shame on psychological distress and its dimensions among Liberian refugees in Nigeria
Source: PLOS Ment Health. 2025 Aug 11;2(8):e0000330. doi: 10.1371/journal.pmen.0000330 (PMC12798500; doi:10.1371/journal.pmen.0000330)
Supplement: S1 File — (PDF) [file pmen.0000330.s002.pdf]

DEMOGRAPHIC DATA

46

Age (in years): ______________________

Gender

______________ Male
______✓_______ Female

Religion

______✓_______ Christian
______________ Muslim
______________ African Traditional Religion

Marital Status

______✓_______ Married
certificate
______________ Divorced
______________ Single
______________ Widowed

Years of Education

______✓_______ Less than Secondary School
______________ Secondary School Certificate
______________ Some College
______________ Bachelor's Degree

Current Country of Residence ______Nigeria______

How long have you lived in your current country of residence (in years and months) ______________

On a scale from 1 to 5 rates the extent to which you identify with the culture of your country of residence

______✓_______ 1 = not at all
______________ 2 = some what
______________ 3 = moderately
______________ 4 = quiet a bit
______________ 5 = completely

What country do you identify as your home? ______Liberia______

Have you ever made an international move during your life (e.g. moved your permanent place of residence from one country to another?)

______________ Yes
______________ No

In what country did you live the longest? ______S______

From what ages did you live there (in years) ______3______ to ______22______

Was your most recent move voluntary?
______________ Yes
______________ No

What was your country of birth? ______Liberia______

How long did you live in your country of birth (in years) ______22______

ESS

Everybody at times can feel embarrassed, self-conscious or ashamed. These questions are about such feelings if they have occurred **at any time in the past year**. There are no 'right' or 'wrong' answers. Please indicate the response which applies to you with a tick.

1. Have you felt ashamed of any of your personal habits?
2. Have you worried about what other people think of any of your personal habits?
3. Have you tried to cover up or conceal any of your personal habits?
4. Have you felt ashamed of your manner with others?
5. Have you worried about what other people think of your manner with others?
6. Have you avoided people because of your manner?
7. Have you felt ashamed of the sort of person you are?
8. Have you worried about what other people think of the sort of person you are?
9. Have you tried to conceal from others the sort of person you are?
10. Have you felt ashamed of your ability to do things?
11. Have you worried about what other people think of your ability to do things?
12. Have you avoided people because of your inability to do things?
13. Do you feel ashamed when you do something wrong?
14. Have you worried about what other people think of you when you do something wrong?
15. Have you tried to cover up or conceal things you felt ashamed of having done?
16. Have you felt ashamed when you said something stupid?
17. Have you worried about what other people think of you when you said something stupid?
18. Have you avoided contact with anyone who knew you said something stupid?
19. Have you felt ashamed when you failed at something that was important to you?
20. Have you worried about what other people think of you when you fail?
21. Have you avoided people who have seen you fail?
22. Have you felt ashamed of your body or any part of it?
23. Have you worried about what other people think of your appearance?
24. Have you avoided looking at yourself in the mirror?
25. Have you wanted to hide of conceal your body or any part of it?

CS = 27
BS = 28
BDS = 7
62

not at all	a little	moderately	very much
( )	( )	( )	( )

Please read each statement and circle a number 0, 1, 2 or 3 which indicates how much the statement applied to you *over the past week*. There are no right or wrong answers. Do not spend too much time on any statement.

The rating scale is as follows:

0 Did not apply to me at all
1 Applied to me to some degree, or some of the time
2 Applied to me a considerable degree, or a good part of the time
3 Applied to me very much, or most of the time

1. I found myself getting upset by quite trivial things 0 1 2 3
2. I was aware of dryness of my mouth 0 1 2 3
3. I couldn't seem to experience any positive feeling at all 0 1 2 3
4. I experienced breathing difficulty (e.g excessively rapid breathing, breathlessness in the absence of physical exertion) 0 1 2 3
5. I just couldn't seem to get going 0 1 2 3
6. I tended to over-react to situations 0 1 2 3
7. I had a feeling of shakiness (e.g legs going to give way) 0 1 2 3
8. I found it difficult to relax 0 1 2 3
9. I found myself in situations that made me so anxious I was most relieved when they ended 0 1 2 3
10. I felt that I had nothing to look forward to 0 1 2 3
11. I found my self getting upset rather easily 0 1 2 3
12. I felt that I was using a lot of nervous energy 0 1 2 3
13. I felt sad and depressed 0 1 2 3
14. I found myself getting inpatient when I was delayed in any way (e.g lifts, traffic lights, being kept waiting) 0 1 2 3
15. I had feelings of faintness 0 1 2 3
16. I felt that I had lost interest in just about everything 0 1 2 3
17. I felt I wasn't worth much as a person 0 1 2 3
18. I felt that I was rather touchy 0 1 2 3
19. I perspired noticeably (e.g hands sweaty) in the absence of high temperatures or physica exertion 0 1 2 3
20. I felt scared with out any good reason 0 1 2 3
21. I felt that life wasn't worth while 0 1 2 3
22. I found it hard to wind down 0 1 2 3
23. I had difficulty in swallowing 0 1 2 3
24. I couldn't seem to get any enjoyment out of the things I did 0 1 2 3
25. I was aware of the action of my heart in the absence of physical exertion (e.g sense of heart rate increase, heart missing a beat) 0 1 2 3
26. I felt down-hearted and blue 0 1 2 3
27. I found that I was very irritable 0 1 2 3
28. I felt I was close to panic 0 1 2 3

29. I found it hard to calm down after something upset me	0 1 2 3
30. I feared that I would be 'thrown' by some trivial but unfamiliar task	0 1 2 3
31. I was unable to become enthusiastic about anything	0 1 2 3
32. I found it difficult to tolerate interruptions to what I was doing	0 1 2 3
33. I was in a state of nervous tension	0 1 2 3
34. I felt I was pretty worthless	0 1 2 3
35. I was intolerant of anything that kept me from getting on with what I was doing	0 1 2 3
36. I felt terrified	0 1 2 3
37. I could see nothing in the future to be hopeful about	0 1 2 3
38. I felt that life was meaningless	0 1 2 3
39. I found myself getting agitated	0 1 2 3
40. I was worried about situations in which I might panic and make a fool of myself	0 1 2 3
41. I experienced trembling (e.g., in the hands)	0 1 2 3
42. I found it difficult to work up the initiative to do things	0 1 2 3

Instructions: We are interested in how you feel about the following statements. Read each statement carefully. Indicate how you feel about each statement by cycling; .

Circle the "1" if you **Very Strongly Disagree**

Circle the "2" if you **Strongly Disagree**

Circle the "3" if you **Mildly Disagree**

Circle the "4" if you are **Neutral**

Circle the "5" if you **Mildly Agree**

Circle the "6" if you **Strongly Agree**

Circle the "7" if you **Very Strongly Agree**

1. There is a special person who is around when I am in need.	1	2	3	4	5	6	7
2. There is a special person with whom I can share joys and sorrows.	1	2	3	4	5	6	7
3. My family really tries to help me.	1	2	3	4	5	6	7
4. I get the emotional help & support I need from my family.	1	2	3	4	5	6	7
5. I have a special person who is a real source of comfort to me.	1	2	3	4	5	6	7
6. My friends really try to help me.	1	2	3	4	5	6	7
7. I can count on my friends when things go wrong.	1	2	3	4	5	6	7
8. I can talk about my problems with my family.	1	2	3	4	5	6	7
9. I have friends with whom I can share my joys and sorrows.	1	2	3	4	5	6	7
10. There is a special person in my	1	2	3	4	5	6	7
life who cares about my feelings.	1	2	3	4	5	6	7
11. My family is willing to help me make decisions.	1	2	3	4	5	6	7
12. I can talk about my problems with my friends.	1	2	3	4	5	6	7
