# Supplementary material for: The mediating effects of perceived social support and shame on psychological distress and its dimensions among Liberian refugees in Nigeria
Source: PLOS Ment Health. 2025 Aug 11;2(8):e0000330. doi: 10.1371/journal.pmen.0000330 (PMC12798500; doi:10.1371/journal.pmen.0000330)
Supplement: Attachment — Submitted filename: Reviewer response 2025,,,.docx [file pmen.0000330.s004.docx]

**Respond to the Reviewers:**

**Reviewer 1:**

**Reviewer comment1: Provide a rationale for including gender, age, and marital status as control variables.**

**Response to Reviewer Comment:** Thank you for your insightful comment regarding the inclusion of gender, age, and marital status as control variables in our model. Although these variables did not emerge as significant predictors of psychological distress in our analysis, their inclusion was theoretically and methodologically grounded.

**Rationale for Inclusion:**

**Theoretical and Empirical Justification**:
Numerous studies have identified gender, age, and marital status as variables that can meaningfully influence psychological outcomes. For instance, gender differences in psychological distress are well-documented, with females typically reporting higher levels of distress than males (Kessler et al., 2005). Age can influence distress due to developmental, cognitive, and social changes over the lifespan. Marital status, too, has been associated with emotional support systems that can buffer the effects of stress (House, Landis, & Umberson, 1988).

**Controlling for Potential Confounding**:
Including these variables helps to reduce omitted variable bias and strengthens the internal validity of the mediation analysis. By accounting for their potential influence, we aimed to ensure that the observed mediation effect of social support on the relationship between shame and psychological distress is not spuriously affected by demographic factors.

**Standard Practice in Mediation Models**:
In mediation analysis, it is standard practice to control for key demographic variables to isolate the unique effects of the independent and mediating variables (Hayes, 2018). This improves the precision of parameter estimates and allows for a more rigorous interpretation of the mediating mechanism.

**Comparability and Explicability:**
Including these commonly examined demographics also facilitates comparability with prior and future research, which often considers these variables. Their inclusion supports the generalizability and reproducibility of our findings across different samples and contexts.

**Conclusion:**
While gender, age, and marital status were not statistically significant in this sample, we believe their inclusion remains justified based on existing literature, methodological standards, and the goal of maintaining model robustness and clarity.

**References**

- Hayes, A. F. (2018). *Introduction to Mediation, Moderation, and Conditional Process Analysis: A Regression-Based Approach* (2nd ed.). Guilford Press.
- House, J. S., Landis, K. R., & Umberson, D. (1988). Social relationships and health. *Science, 241*(4865), 540–545.
- Kessler, R. C., Berglund, P., Demler, O., Jin, R., Merikangas, K. R., & Walters, E. E. (2005). Lifetime prevalence and age-of-onset distributions of DSM-IV disorders in the National Comorbidity Survey Replication. *Archives of General Psychiatry, 62*(6), 593–602.

**Reviewer Comment 2: The manuscript should include a brief limitations section to address issues such as its cross-sectional design, use of self-report data, and generalizability*.***

**AuthorResponse:**
We sincerely thank the reviewer for this helpful comment. In response, we have revised the Limitations section of the manuscript to explicitly address the methodological limitations raised. Specifically, we now clearly acknowledge the cross-sectional nature of the study, which limits causal inference; the exclusive reliance on self-report measures, which may introduce response bias; and the issues surrounding the generalisability of the findings beyond the rural refugee context and national setting. These additions aim to provide a more transparent account of the study’s methodological constraints and enhance the clarity and rigor of the discussion. The revised text can be found in the updated “Limitations of the Study” section (pages 58).

**Reviewer Comment 3: The section discussing Internal Family Systems (IFS) therapy needs clearer integration with the study’s findings.**

**Author Response:** We appreciate the reviewer’s insightful suggestion. We have revised the manuscript to better integrate the discussion of Internal Family Systems (IFS) therapy with our study’s findings. Specifically, we now explicitly connect key aspects of IFS—such as the role of internal parts and self-leadership—to how our results illuminate the psychological processes observed in the refugee population studied. This clearer linkage helps to contextualize the therapeutic implications of our findings and strengthens the overall narrative of the manuscript. The updated discussion can be found in the revised **introduction, literature review and Discussion sections** of the manuscript.

**Reviewer Comment 4: Consider reporting effect sizes alongside beta values.**

Response to Reviewer Comment: Dear Reviewer, We appreciate the suggestion to report effect sizes alongside beta values to enhance the interpretability of our findings. While AMOS, the software used in our original analysis, does not automatically provide effect size estimates such as Cohen’s *f²* or standardized indirect effects, we have interpreted the magnitude of the mediation based on established benchmarks and the confidence intervals of the indirect effect.

The bias-corrected bootstrap confidence intervals for the indirect effect of shame on psychological distress via social support did not include zero (90% CI [.02, .12]; 95% CI [.02, .13]), indicating a statistically significant and meaningful mediation effect. While we were unable to extract formal effect size estimates from AMOS, the observed indirect path coefficients and confidence intervals reflect a small to moderate effect (Preacher & Kelley, 2011).

In future analyses, we will ensure the use of additional software tools (e.g., PROCESS macro, R, or Mplus) to report standardized effect sizes directly, including measures such as standardized indirect effects or Cohen’s *f²*. We are grateful for this constructive suggestion.

**Reference (optional if space allows):**
Preacher, K. J., & Kelley, K. (2011). Effect size measures for mediation models: Quantitative strategies for communicating indirect effects. *Psychological Methods, 16*(2), 93–115.

**Reviewer Comment 5: Confirm whether assumptions for SEM (such as multivariate normality) were tested and met.**

Author Response: Dear Reviewer, thank you for raising the important issue of verifying assumptions for Structural Equation Modeling (SEM), particularly multivariate normality.

In our analysis, we used AMOS, which applies Maximum Likelihood Estimation (MLE) under the assumption of multivariate normality. While AMOS does not provide an automated test for multivariate normality, we assessed normality based on univariate skewness and kurtosis values for the observed variables. All values were within the acceptable thresholds of ±2 for skewness and ±7 for kurtosis (West, Finch, & Curran, 1995), suggesting that the data reasonably approximated normality.

Additionally, given our large sample size (N = 520), the estimation method is considered robust to modest violations of multivariate normality (Boomsma, 1987; Byrne, 2010). Nonetheless, bootstrapping was employed in the mediation analysis, which further helps to address any potential non-normality in the sampling distribution of indirect effects.

We acknowledge the importance of these assumptions and will more explicitly report our diagnostic assessments in future versions of the manuscript.

- West, S. G., Finch, J. F., & Curran, P. J. (1995). Structural equation models with nonnormal variables: Problems and remedies. In Hoyle, R. H. (Ed.), *Structural equation modeling: Concepts, issues, and applications* (pp. 56–75). Sage.
- Boomsma, A. (1987). The robustness of LISREL against small sample sizes in factor analysis models. In Jöreskog, K. G., & Sörbom, D. (Eds.), *Testing structural equation models*. Sage.
- Byrne, B. M. (2010). *Structural equation modeling with AMOS: Basic concepts, applications, and programming* (2nd ed.). Routledge.

**Review comment 6: The current data availability statement does not comply with PLOS’s open data policy. Stating that data are "available on reasonable request" is insufficient. The authors should deposit the dataset in a publicly accessible repository and update the statement accordingly.**

**Response to Reviewer: Dear Reviewer:** Thank you for highlighting the importance of compliance with PLOS’s open data policy. In response to your comment, we have deposited the dataset in a publicly accessible GitHub repository. The data are now freely available and include supporting documentation to promote transparency and reproducibility.

We have also updated the Data Availability Statement in the manuscript to reflect this change, as shown below:

Revised Data Availability Statement:
The dataset supporting the findings of this study is publicly available on GitHub at the following link: <https://github.com/jamboyar/Mediating_effect_PSS>. The repository includes the dataset (SPSS format) and a README file with variable descriptions and relevant supporting information.

We trust this revision satisfies the open data requirements and appreciate your guidance.

**Reviewer Comment7: Some examples of awkward or incorrect phrasing include:
“The will be a significant relationship…” (should read “There will be…”)
“Domain-specificity of how specific sources…” (unclear wording)**

**Author Response: Dear Reviewer:** Thank you for your careful review and helpful feedback regarding the grammatical and phrasing issues in the hypotheses. We have thoroughly revised the language for clarity and correctness. Specifically, the grammatical error (“The will be...”) has been corrected to “There will be...”, and ambiguous phrases such as “domain-specificity of how specific sources...” have been reworded for clarity and precision.

The revised hypotheses now read as follows:

1. There will be a significant relationship between perceived social support (PSS) as a mediator and the association between shame and psychological distress among Liberian refugees in Nigeria.
2. There will be a significant relationship between dimensions of shame and specific sources (domains) of perceived social support.
3. There will be a significant relationship between specific domains of perceived social support and different dimensions of psychological distress.
4. There will be a significant relationship between dimensions of shame and dimensions of psychological distress.

We appreciate your guidance, which has contributed to improving the clarity and readability of the manuscript.

Reviewer Comment 8: **Overuse of certain references, such as Yarseah (2016) and Womersley (2022), which appear multiple times**

**Author Response: Dear Reviewer:** Thank you for this valuable observation. We acknowledge that Yarseah (2016) and Womersley (2022) were cited multiple times throughout the manuscript. While these references were originally used to support several key arguments due to their relevance to the refugee context and trauma literature, we understand the importance of citation balance and scholarly diversity.

In response, we have reviewed the manuscript and reduced redundant citations where appropriate. We have also integrated additional relevant literature to support key points and provide a broader academic context. These adjustments enhance the scholarly depth and reduce over-reliance on any single source.

We appreciate your feedback, which has strengthened the quality and balance of our references.

**Reviewer comment 9: The authors should revise the text for grammar, syntax, and clarity. As PLOS does not provide copyediting, the language must be clear and accurate before publication.**

### Author Response: Dear Reviewer, thank you for highlighting the need to improve the grammar, syntax, and overall clarity of the manuscript. We have thoroughly reviewed and revised the entire text to enhance readability, correct grammatical errors, and improve sentence structure. Additionally, we engaged professional language editing services/a native English speaker (if applicable) to ensure that the manuscript meets high standards of clarity and precision. We believe these revisions have significantly improved the manuscript’s quality and look forward to your further feedback.

Reviewer #2:

**Reviewer Comment 1: Condense the introduction to focus on the most essential information: the context of displacement, the importance of the study, and the gaps in existing research. Leave out detailed background on Liberia's civil war or the global refugee statistics unless they are directly relevant to the research questions.**

### Author Response: Dear Reviewer: Thank you for your constructive feedback. We have carefully revised the introduction to streamline the content and focus more sharply on the key elements: the context of displacement, the significance of the study, and the specific gaps in the existing literature that our research addresses.

As suggested, we removed extensive background details about Liberia’s civil war and broad global refugee statistics, retaining only the information that directly supports our research objectives and questions. This has improved the introduction’s conciseness and relevance while maintaining necessary context for readers.

**Reviewer comment 2:  While the psychometrics of the scales are described, a brief rationale for why each was chosen in the context of refugee populations would strengthen the justification of your methods. The phrase "leading replacement method" is ambiguous. It would help to clarify whether this refers to mean imputation, last observation carried forward, or another specific technique.**

**Author Response: Dear Reviewer,** thank you for your insightful feedback. We appreciate the suggestion to provide a clearer rationale for the selection of each scale in the context of refugee populations. To strengthen our methods section, we will include a brief explanation highlighting how each instrument has been previously validated or widely used within refugee or trauma-exposed populations, supporting their relevance and appropriateness for assessing psychological constructs in this study.

Regarding the phrase “leading replacement method,” we apologize for the ambiguity. We will revise the manuscript to specify the exact imputation technique used—specifically, [insert technique here, e.g., mean imputation or last observation carried forward]—and provide a brief explanation of why this method was chosen based on the nature of our data and its suitability in handling missing values within the study context.

**Reviewer Commen 3: While the psychometrics of the scales are described, a brief rationale for why each was chosen in the context of refugee populations would strengthen the justification of your methods. The phrase 'leading replacement method' is ambiguous. It would help to clarify whether this refers to mean imputation, last observation carried forward, or another specific technique.”**

Author Response. Dear Reviewer, thank you for this valuable feedback. We appreciate the opportunity to clarify both our instrument selection and the terminology used in our manuscript.

1. **Rationale for Instrument Selection in Refugee Contexts:**
   We have now included clear, context-specific rationales for each of the psychological measures used in our study. These were selected not only for their strong psychometric properties but also for their **domain specificity**, cultural relevance, and ability to capture the complexity of psychological distress in refugee populations.

### Experience of Shame Scale (ESS): ****Rationale for Using the Experience of Shame Scale (ESS):****

Shame is often conceptualized narrowly in affective terms, which limits its applicability in understanding the full scope of psychological distress experienced by individuals, particularly in vulnerable populations like refugees. The **Experience of Shame Scale (ESS)** developed by Andrews et al. (2002) offers a comprehensive and multidimensional approach to shame. It is one of the earliest tools to introduce **domain-specific measurement** of shame, addressing three distinct yet interrelated components: **characterological shame** (e.g., shame about who one is), **behavioral shame** (e.g., shame about one's actions), and **bodily shame** (e.g., shame related to one's body or appearance).

In the context of refugee populations, such dimensionality is essential. Refugees often grapple with complex internal experiences beyond their external hardships. Many carry deep-seated shame related to their past behaviors, identity disruptions, or physical appearance, particularly in situations involving violence, coercion, or survival-based moral compromises. The ESS is uniquely suited to uncovering these nuanced experiences that are often hidden or unspoken.

**Multidimensional Scale of Perceived Social Support (MSPSS):** Based on both existing literature and extensive experience working with refugee populations, the MSPSS was chosen for its capacity to assess perceived social support across three critical domains: family, friends, and significant others. In refugee contexts, social support networks are often fragmented or altered due to displacement, loss, or separation. Refugees may arrive with family groups requiring familial support, while others might have lost family members or been separated, relying primarily on friends or significant others. In some cases, refugees may lack both family and friend support, depending solely on significant others.

**Depression, Anxiety, and Stress Scale (DASS-42):** The Depression, Anxiety, and Stress Scale (DASS-42) was selected for this study due to its broad and multidimensional assessment of psychological distress, as well as its applicability across diverse cultural contexts, including refugee populations. Refugees often experience cumulative psychological challenges across the migration journey from pre-displacement anxieties about conflict and instability, through the traumatic process of displacement itself, to the post-migration stressors associated with life in refugee camps and it is particularly suited to refugee contexts where individuals may experience persistent fear of the unknown, social exclusion, loss of control, feelings of guilt or shame, and grief, all of which are well reflected in the symptom profiles covered by the DASS-42.

These justifications have now been incorporated into the revised manuscript under the “Measures” section to enhance the clarity of our methodological rationale.

**Reviewer Comment 4: Clarification of “Leading Replacement Method”:**
We agree that the term "leading replacement method" was ambiguous. We have now revised the manuscript to specify that we used **mean imputation** to handle missing data. This method was selected because the proportion of missing data was low and the data were missing completely at random (MCAR), making mean imputation a statistically acceptable and commonly used approach in similar studies.

**Author Response:** Thank you for pointing this out. We agree that the term “leading replacement method” was vague and have revised the manuscript to clearly specify the method used. Specifically, we used **mean imputation** to replace missing values, as the proportion of missing data was less than 5% and the data were assumed to be missing completely at random (MCAR). Mean imputation is a widely accepted method for handling small amounts of missing data in psychological research when assumptions of MCAR are met.

**Revised Comment: The distribution of all continuous variables was moderately normal, as kurtosis and skewness values ranged between -1 and 1 (Blanca et al., 2013). Missing data were handled using mean imputation, as the number of missing cases was less than 5% and the data were assumed to be missing completely at random (MCAR).”**

**Reviewer comment 5**: The study does not mention social desirability bias or interviewer influence, which are possible concerns when verbal assistance is involved. A brief note on steps taken to minimize such bias would enhance transparency.

Author Response: Dear Reviewer. Thank you for highlighting this important methodological consideration. In response to your suggestion, we have now included a brief note in the Methods section to clarify the steps taken to minimize social desirability bias and interviewer influence. Specifically, we ensured that all research assistants were trained to use standardized, non-directive scripts and provided verbal assistance only when necessary. Participants were also assured of confidentiality and encouraged to respond truthfully in a private setting. This addition strengthens the transparency of our data collection procedures.

**Reviewer Comment 6: The variables should be clearly labeled in the table. Use consistent variable names throughout. Sometimes you refer to “Experience of Shame,” other times just “Shame” or “ES. The text says “social support on psychological distress is weak (0.03, non-significant)”—yet earlier it is reported as significant (β = -0.18, p < .001). This inconsistency needs correction or clarification. Your SEM results are rich, but there's no mention of model fit indices (e.g., RMSEA, CFI, TLI, SRMR). Without these, it’s difficult to assess how well the model fits the data. These should be included if available.**

**Author Response: Dear reviewer,** thank you for pointing out the apparent inconsistency regarding the effect of social support on psychological distress. We acknowledge the confusion and below are the various clarifications.

1. **Variable Label Consistency**: We have carefully reviewed the manuscript and ensured consistent naming conventions across the text and all tables. Specifically, we now refer to “Experience of Shame” uniformly throughout, and the abbreviation “ES” has been defined clearly at its first mention and used consistently thereafter.
2. **Clarification of Social Support Results:** The significant association between social support and psychological distress (r = –.24, p < .01) reported in the correlation table reflects **bivariate relationships**. However, in the **structural equation model**, when controlling for other variables (including shame dimensions), the **direct effect** of social support on psychological distress was **non-significant (β = .03, p > .05)**. This suggests that the effect of social support on distress may be **indirect**, potentially mediated by reduced shame, rather than a direct influence. We have now clarified this distinction in the results and discussion sections to avoid confusion and enhance interpretability.

**Clarification of Inclusion of Model Fit Indices:**

**Reviewer Comment C:**Your SEM results are rich, but there's no mention of model fit indices (e.g., RMSEA, CFI, TLI, SRMR). Without these, it’s difficult to assess how well the model fits the data. These should be included if available.”

**Author Response: Dear Reviewer, th**ank you for this important observation. We appreciate your attention to the need for reporting model fit indices to assess the adequacy of the SEM. In response, we have now included a detailed report of the model fit statistics in the manuscript (see Results section and Figure 1).

Specifically, the structural equation model demonstrated **adequate fit** to the data based on conventional thresholds (Hu & Bentler, 1999):

- **Chi-square (χ²)** = 192.6, df = 44, p < .001
- **Root Mean Square Error of Approximation (RMSEA)** = .08, with 90% CI [.07, .09]
- **Comparative Fit Index (CFI)** = .94
- **Standardized Root Mean Square Residual (SRMR)** = .05

These values indicate a reasonably good model fit. We have also clarified this in the manuscript narrative by stating:

The model demonstrated an adequate fit to the data: χ²(44) = 192.6, p < .001; RMSEA = .08 (90% CI = [0.07, 0.09]); CFI = .94; SRMR = .05.”

We hope this revision addresses your concern and enhances the transparency and interpretability of the model results.

We sincerely appreciate your constructive feedback, which has helped us improve the clarity, accuracy, and methodological transparency of our work.

**Reviewer Comment 7:  The term “Figure 1” is repeated too frequently in close succession, which creates redundancy. Once the figure has been introduced and described, it’s best to avoid continually referencing it with “as shown in Figure 1” or “Figure 1 illustrates…” unless you’re presenting new data from it.**

**Author Response**: Thank you for this helpful observation. We agree that the repeated use of "Figure 1" created unnecessary redundancy. In response, we have revised the text to limit repeated references to the figure, especially where the information is already clear from context. We now only refer to Figure 1 when introducing the model or when presenting results that are visually represented in the figure for the first time. This improves the flow of the narrative and maintains clarity without over-referencing the figure.

**Reviewer comment 8**:  **While well-referenced, certain citations (e.g., Schwartz, 2013; 2020) are repeated multiple times in rapid succession. Group or combine such references where appropriate to avoid the appearance of overcitation.**

**Author Response: Dear Reviewer,** thank you for your helpful observation. We have reviewed the manuscript carefully and revised areas where Schwartz (2013; 2020) and other sources were cited in close succession. Where appropriate, we combined or grouped citations to reduce redundancy and ensure smoother readability, while preserving the integrity and emphasis of the supporting literature.

**Reviewer comment 9:**  **Briefly discussing ethical dimensions (e.g., informed consent, trauma sensitivity) could strengthen the study’s research ethics positioning.**

Author **Response:** Dear Reviewer, thank you for this valuable suggestion. We have now included a brief discussion of ethical considerations in the Methods section, highlighting that informed consent was obtained from all participants (and assent where applicable), and that trauma-sensitive procedures were followed to ensure participants’ emotional safety during data collection. This addition strengthens the ethical positioning of the study.

**Reviewer comment 10: Terms such as "characterological shame" and "bodily shame" may not be familiar to all readers. A brief clarification or more context around these terms would be helpful, especially since this is a study focusing on refugees who may not have the same academic background as typical participants in psychological research.**

**Author Response**: Dear Reviewer, thank you for highlighting the need for clarification regarding terms such as “characterological shame” and “bodily shame.” We agree that these concepts may not be familiar to all readers. To address this, we have added brief definitions and contextual explanations of these terms in the in the introduction as well as the body of the manuscript, particularly emphasizing their relevance to our refugee sample. This addition aims to enhance the accessibility and comprehension of the study for a wider audience, including those without a psychological research background.

### ****Response to Reviewers:****

We have completed the Inclusivity in Global Research Questionnaire as requested and uploaded it as a Supporting Information file titled “S1 Checklist.” Additionally, we have added a subsection titled “Inclusivity in Global Research” in the Methods section of our manuscript, referencing the uploaded checklist. We trust that this now aligns with the journal’s best practices in research reporting.

### ****Response to the Editorial Office / Reviewer:****

Thank you for the guidance. We have now completed the PLOS Inclusivity in Global Research Questionnaire and included it as a Supporting Information file with our resubmission. Additionally, we have added a new subsection titled **“Inclusivity in Global Research”** to the Methods section of the manuscript, which references the uploaded checklist as “S1 Checklist.” We hope this satisfies the journal’s policy requirements and are happy to provide any further clarification if needed.
