# Supplementary material for: The mediating effects of perceived social support and shame on psychological distress and its dimensions among Liberian refugees in Nigeria
Source: PLOS Ment Health. 2025 Aug 11;2(8):e0000330. doi: 10.1371/journal.pmen.0000330 (PMC12798500; doi:10.1371/journal.pmen.0000330)
Supplement: Attachment — Submitted filename: Rebuttal Letter June.docx [file pmen.0000330.s006.docx]

**Response to the Reviewer’s Comments**

***Manuscript name: Mediating Effects of Perceived Social Support and Shame on Psychological Distress and Its Dimensions among Liberian Refugees in Nigeria***

**Manuscript ID: *PMEN-D-25-00158R1***

**Corresponding Author: *Dogbahgen Alphonso Yarseah***

We sincerely thank the reviewers and editorial team for their thoughtful and constructive feedback. Below, we provide detailed responses to each comment and describe the corresponding revisions made to the manuscript. All changes are highlighted in the revised version.

**Reviewer comment 1:**

*The manuscript would benefit from thorough copyediting to address typographical and grammatical errors."*

**Response:**
Thank you for this valuable feedback. We sincerely acknowledge the presence of typographical and grammatical errors in the original submission. In response, we have carefully and thoroughly revised the entire manuscript for clarity, grammar, punctuation, and style. The revised version has also undergone professional copyediting to ensure it meets the highest language standards. We believe these improvements have significantly enhanced the overall readability and quality of the manuscript.

**Reviewer comment 2**:

Several phrases are awkward or imprecise (e.g., "Table 3 displaced" should be "displays").

**Response**:

Thank you for pointing this out. We have carefully reviewed the manuscript for awkward or imprecise phrasing and corrected the specific instance noted (e.g., “Table 3 displaced” has been revised to “Table 3 displays”). Additionally, we conducted a thorough edit of the manuscript to improve clarity, precision, and readability across all sections. We believe these revisions have enhanced the overall linguistic quality of the manuscript.

**Reviewer comment 3:**

*There are copy-paste artifacts that may cause confusion (e.g., a reference to “suicidal PTSD” in Table 4 that is not applicable to this study).*

**Response:**

. Thank you for drawing our attention to this error. The title of Table 4 mistakenly referred to “the indirect effect of psychological inflexibility on suicidal PTSD,” which was inadvertently carried over from an earlier draft. The correct mediation pathway, as presented in the table, is from **experience of shame to psychological distress through social support**.
We have corrected the table title accordingly and have thoroughly reviewed the entire manuscript to ensure that all copy-paste inconsistencies have been resolved.

**Reviewer Comment 4:**
*The analysis is robust, but further clarity around the non-significant direct path from social support to distress would strengthen interpretation.*

**Author Response:**
Thank you for this thoughtful comment. In response, we have added a clarification in the Results and Discussion sections of the revised manuscript (see page 37). Specifically, we now note that although the **direct path from perceived social support to psychological distress was not statistically significant (β = 0.03, p > .05)**, the **indirect effect of shame on distress through social support was significant (β = 0.06; 95% CI [0.02, 0.13])**. This suggests that perceived social support may not independently predict psychological distress but rather functions as a **mediating mechanism** in the relationship between shame and distress. We have also expanded the theoretical explanation to highlight that emotional states like shame may influence how social support is internalized and utilized, particularly in refugee contexts where self-conscious emotions are prominent.

**Reviewer Comment 5:**
*Consider acknowledging the possibility of alternative model specifications (e.g., moderated mediation), as you briefly mention in your discussion.*

**Author Response:**
Thank you for this valuable suggestion. We agree that considering alternative model specifications, such as **moderated mediation**, would enrich the interpretation of our findings. In response, we have revised the **Limitations section** of the manuscript (see page 60, paragraph ) to explicitly acknowledge the potential for moderated mediation models. We now note that factors such as gender, age, or duration of displacement could influence the strength of the indirect effects, and future research should examine these possibilities. This addition reinforces our recognition of the complex interplay between social and emotional processes in refugee mental health and highlights important directions for further study.

### ****Reviewer Comment 6: Figures and Tables****

Ensure that all figures (particularly Figure 1 and Figure 2) are present, legible, and clearly described. Currently, figure placement and description are insufficient for full appraisal.

**Author Response:**
Thank you for this helpful observation. We would like to clarify that the manuscript contains **only one figure**, now correctly labeled as **Figure 1**. In response to your feedback, we have:

- Enhanced the **clarity, formatting, and resolution** of Figure 1 for improved legibility.
- Updated the **figure legend** to clearly describe all constructs, indicators, and path relationships.
- Provided a more detailed **interpretation in the Results section**, clarifying the mediating and non-significant paths (particularly the indirect role of social support).

**Reviewer Comment 7:**
*Limitations: Consider reinforcing the limitations related to self-report bias and the potential sensitivity of constructs like shame and distress in post-conflict refugee contexts.*

**Response:**
Thank you for this valuable suggestion. We have now strengthened the limitations section to explicitly acknowledge the potential for self-report bias, particularly in relation to emotionally sensitive constructs such as shame and psychological distress. Given the post-conflict refugee context, we recognize that some participants may have underreported or altered their responses due to social desirability, fear of stigma, or emotional discomfort. These factors may influence the accuracy or depth of reported experiences. We have added this clarification to the revised manuscript to provide a more balanced interpretation of our findings.

**Journal Requirement:**

“Please review your reference list … If you have cited papers that have been retracted, please include the rationale … or remove these references … Any changes to the reference list should be mentioned in the rebuttal letter …”

**Author’s Response:**

Dear Editor / Editor’s Editorial Team,

Thank you for your guidance regarding the reference list. We have thoroughly reviewed the reference list and can confirm that none of the cited sources are retracted. We have removed several references that were inadvertently retained from earlier drafts, and added new citations that were missing (marked in red in the revised manuscript). All entries have been updated to conform with APA 7th edition formatting. We believe this enhances the manuscript’s accuracy and comprehensiveness.

Following your recommendation, we have thoroughly reviewed and revised our reference section. Below is a summary of the actions taken:

**1. Missing References Added**

We have now included the following references, which were previously missing. Each has been cited appropriately within the manuscript and highlighted in **red** for editorial clarity:

1. **Bunn et al. (2022)** – *Family-based mental health interventions for refugees across the migration continuum: A systematic review.*
2. **de Hooge et al. (2018)** – *The social side of shame: Approach versus withdrawal.*
3. **Doran & Lewis (2012)** – *Components of shame and eating disturbance among clinical and non-clinical populations.*
4. **Farr et al. (2021)** – *Early shaming experiences and psychological distress: The role of experiential avoidance and self-compassion.*
5. **Gilbert (1998)** – *What is shame? Some core issues and controversies.*
6. **Hejdenberg & Andrews (2011)** – *The relationship between shame and different types of anger: A theory-based investigation.*
7. **Ioannou et al. (2019)** – *Coping with depressive symptoms in young adults: Perceived social support protects against depressive symptoms only under moderate levels of stress.*
8. **Kondrat et al. (2017)** – *The mediating effect of social support on the relationship between the impact of experienced stigma and mental health.*
9. **Kaniasty & Norris (1993)** – *A test of the social support deterioration model in the context of natural disaster.*
10. **Lawley & Tompkins (2013)** – *Metaphors in mind: Transformation through symbolic modelling.*
11. **Liu et al. (2020)** – *Factors associated with depression, anxiety, and PTSD symptomatology during the COVID-19 pandemic: Clinical implications for U.S. young adult mental health.*
12. **Leeming & Boyle (2004)** – *Shame as a social phenomenon: A critical analysis of the concept of dispositional shame.*
13. **Matos et al. (2012)** – *Above and beyond emotional valence: The unique contribution of central and traumatic shame memories to psychopathology vulnerability.*
14. **Porter & Haslam (2005)** – *Predisplacement and postdisplacement factors associated with mental health of refugees and internally displaced persons: A meta-analysis.*
15. **Sampogna et al. (2012)** – *Living with psoriasis: Prevalence of shame, anger, worry, and problems in daily activities and social life.*
16. **Schweitzer et al. (2006)** – *Trauma, post-migration living difficulties, and social support as predictors of psychological adjustment in resettled Sudanese refugees.*
17. **Swan & Andrews (2003)** – *The relationship between shame, eating disorders and disclosure in treatment.*
18. **Tylka & Hill (2004)** – *Objectification theory as it relates to disordered eating among college women.*

**2. Incorrect or Irrelevant References Removed or Replaced**

- *Kröner & Müller (2022)* was **removed** as it no longer aligns with the revised focus of the manuscript.
- Other citations were corrected where previously misattributed, such as the mislabeling of *Johnson et al. (2014)* as *Jones et al.*

**3. Replaced with More Relevant References**

- *Oren-Yagoda et al. (2024)* – *Gender differences in shame among individuals with social anxiety disorder.*
- *Strøm et al. (2018)* – *The mediating role of shame in the relationship between childhood bullying victimization and adult psychosocial adjustment.*

.

**4. No Retracted References Present**

A full audit of the reference list confirms that no retracted articles are cited in the manuscript. If, in future revisions, a retracted source must be included, we will clearly identify its status and cite the retraction notice as per your guidelines.

**5. APA 7th Edition Formatting Ensured**

All references have been updated to conform with APA 7th edition requirements, including capitalization, italicization, punctuation, DOIs, and proper structure.

**Closing Note:**

We deeply appreciate the reviewer’s time and valuable input, which helped strengthen the clarity, depth, and theoretical contributions of the manuscript. We hope the revisions meet the expectations of the editorial team and reviewers.

Thank you once again for the opportunity to revise and improve our work.

Sincerely,
**Dogbahgen Alphonso Yarseah**
Corresponding Author
On behalf of all co-authors
